# Responses and coping methods of different testicular cell types to heat stress: overview and perspectives

**DOI:** 10.1042/BSR20210443

**Published:** 2021-06-16

**Authors:** Hui Cai, Dezhe Qin, Sha Peng

**Affiliations:** College of Veterinary Medicine, Shaanxi Centre of Stem Cells Engineering and Technology, Northwest A&F University, Yangling, Shaanxi 712100, P.R. China

**Keywords:** heat stress, Leydig cells, reproduction, spermatocytes, spermatogonial stem cells

## Abstract

To facilitate temperature adjustments, the testicles are located outside the body cavity. In most mammals, the temperature of the testes is lower than the body temperature to ensure the normal progression of spermatogenesis. Rising temperatures affect spermatogenesis and eventually lead to a decline in male fertility or even infertility. However, the testes are composed of different cell types, including spermatogonial stem cells (SSCs), spermatocytes, spermatozoa, Leydig cells, and Sertoli cells, which have different cellular responses to heat stress. Recent studies have shown that using different drugs can relieve heat stress-induced reproductive damage by regulating different signaling pathways. Here, we review the mechanisms by which heat stress damages different cells in testes and possible treatments.

As global temperatures rise, heat stress has become one of the most important factors affecting the reproductive performance of mammals. The testicular temperature of most male animals is lower than their body temperature. Excessive heat affects spermatogenesis in the testis, reduces the quality and quantity of sperm, and even causes infertility. The testes contain different cell types. Heat stress has different effects on these cells, such as apoptosis, DNA damage, blood–testis barrier (BTB) disruption, and hormone secretion disorders. Recent research has shown that different treatment strategies should be used for testicular heat stress. This review discusses the effects of and molecular responses to heat stress in different cells of the testes and summarizes effective therapies.

## The influence of heat stress on male reproduction

Male gamete generation involves spermatogenesis in the testicular seminiferous tubules and subsequent sperm maturation in the epididymis. Human spermatogenesis takes ∼74 days to complete one cycle, and sperm in the epididymis mature in ∼12 days. Sequential cellular events during spermatogenesis begin in the basal compartment and end in the apical region of the seminiferous tubules [1]. Mammalian spermatogenesis is divided into mitosis, meiosis, and spermiogenesis. In mitosis, spermatogonia divide into two spermatocytes, and spermatocytes produce four sperms by meiosis. In spermiogenesis, the round spermatids undergo morphological changes to eventually form sperm [2]. After leaving the seminiferous tubules, the sperms concentrate in the epididymis head; they then mature in the epididymis body and are stored in the tail [3].

The normal spermatogenic process needs the lower temperature in scrotum than in body. Part of infertile men have symptoms that their temperature of scrotum is higher than that in males with normal reproductive function, and their sperm will lose qualities gradually as the temperature increases [4]. A degree of heating will cause the stress in testis in many species. Previous studies have confirmed the finding. Heat stress decreases the sperm motility or even density, therefore increases sperm deformity and the infertility rate among boars [5]. In Brangus bulls, similarly, semen quality and the seminal plasma proteome are also changed because of thermal stimulation [6]. Heat stress induces dysplasia in dairy cattle and a reduction in sperm quality and libido in bulls. Persistent heat stress can significantly alter blood physiological and biochemical signals in male Hainan black goat, and reduce serum reproductive hormone levels and semen quality. To sum up, heat stress has a great impact on the density and quality of sperm. Increased scrotum temperature causes testicular hypoplasia and stagnant spermatogenesis [7] and decreases inhibin B (a biochemical marker of spermatogenesis) [8], reducing the number of sperm and eventually causing infertility [9]. The main effects of heat stress on cells are changes in membrane fluidity and cell morphology and damage to the ultrastructure of mitochondria and the nucleus [10]. Depending on the duration and strength of heat stress, cell death may also occur [11,12].

## Effects of heat stress on different cells of the testes

**Figure 1:**
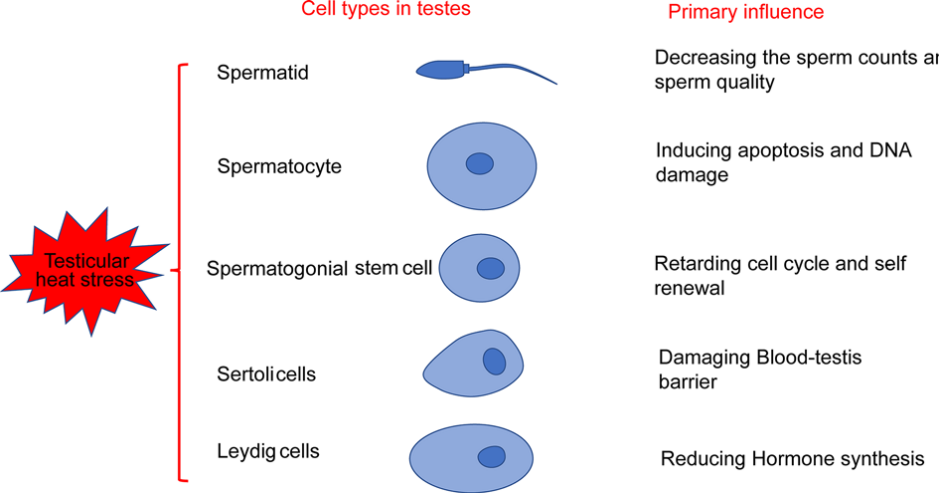
Effects of heat stress in the testes Heat stress can affect most cell types in the testis, including spermatids, spermatocytes, spermatogonial stem cells, Sertoli cells, and Leydig cells. However, heat stress has a variety of impacts on these cell types.

Spermatogenic cells are more susceptible to heat stress damage than other cells because of their frequent division and lack of superoxide dismutase [4,13,14]. However, when undergoing heat stress, the sensitivity, response, and physiological and pathological changes of different types of cells in the testes differ (Figure 1). Many studies have shown that spermatocytes and mature sperm cells are sensitive to changes in temperature. The most vulnerable cells to heat damage in humans and rats are the zygotene and pachytene spermatocytes and early round spermatozoa [15]. Increased testicular temperature has also been reported to interfere with the function and morphology of Sertoli cells and disrupt the tight connection between cells and the BTB. Heat stress of Leydig cells mainly effects the hormone secretion of Leydig cells [16].

### Apoptosis and DNA damage in spermatocytes

Spermatocytes are produced by spermatogonium differentiation. The first generation of spermatogonium, primitive type A spermatogonium, are produced on several days following birth. Part of primitive type A spermatogonium differentiate into type A spermatogonium, and the rest of them maintain the quantity of themselves through mitosis. Then, type A spermatogonium differentiate into type B spermatogonium. Type B spermatogonium enter the meiosis stage after mitosis, and the primary spermatocytes appear; they leave the basement membrane and enter the spermatogenic epithelium through the tight junctions of the epithelial cells and begin meiosis. The spermatocytes form round sperm cells after undergoing two meiotic divisions, during which the spermatocytes are prone to apoptosis induced by external temperature stimulation, which affects mammalian fertility [17]. A quantitative analysis of mice showed the most sensitive germ cells stages were pachytene spermatocytes and round spermatids [18,19], the result is confirmed in humans [20] and rats [21].

**Figure 2:**
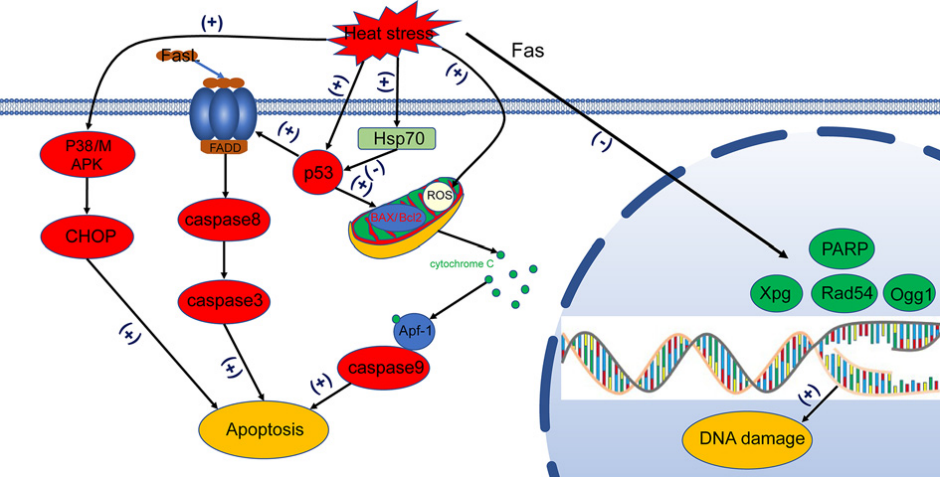
Apoptosis pathways in spermatocytes Heat stress can induce spermatocyte damage. On one hand, BAX (pro-apoptotic protein) responds to heat stress and accumulates in the mitochondria, while BCL-2 is phosphorylated and loses activity. BAX is integrated into the outer mitochondrial membrane, causing a conformational change that releases cytochrome *c* into the cytoplasm. Cytochrome *c* interacts with APAF-1 to form a complex that activates the caspase cascade. On the other hand, heat stress connects the death receptor FAS to its ligand FASL through p53. FAS recruits FAS-related death domain (FADD) through the shared death domain (DD) to form a complex, which is bound to the caspase-8 promoter, triggering the caspase cascade. Heat stress activates spermatocyte apoptosis by directly activating the p38-MAPK signaling pathway independently of p53 signals. Finally, heat stress also inhibits DNA repair-related genes, such as *Ogg1* (involved in base excision repair), *Xpg* (involved in nucleotide excision repair), *Rad51* and *Rad54* (involved in double-strand break repair) and, eventually, spermatocyte replication and meiosis separation, resulting in reproductive damage. p53 signaling plays a very important role in these genes and signaling pathways. p53 causes cell death through FAS; it also disrupts the BCL-2/BAX balance and triggers mitochondria-related apoptosis.

Heat stress primarily activates the classical apoptotic pathway (mitochondrial apoptotic pathway) in spermatocytes and sperm cells [21,22] (Figure 2). The change in the mitochondrial transmembrane potential is related to many mechanisms. Green et al. reported that membrane permeability transition pore (PTP) is located between the inner and outer mitochondrial membranes and involves a complex of proteins [23]. When a cell is apoptotic, the PT pore is opened, cytochrome *c* is released, which combines with Apaf-1 and activates the caspase system, finally resulting in apoptosis [24]. Studies have shown that BAX (BCL2-associated X) transfers from the cytoplasm to the nucleus of spermatocytes after heat shock, and redistribution is completed, while BCL-2 expression does not significantly change [25]. Distorting the ratio of BAX and BCL-2 induces apoptosis; while, opening mitochondrial PT pores releases cytochrome *c* [26]. Releasing cytochrome *c* forms a complex with apoptotic protease activating factor 1 (APAF-1) [27,28]. The complex combines with caspase 9 and activates the caspase cascade via executioner caspases like caspases-3, -6, and -7 [29]. Additionally, p53-dependent or -independent pathways and p38 mitogen-activated protein kinase (MAPK)-signaling activation are involved in mitochondria-mediated apoptosis of spermatogenic cells [30]. A similar process has also been found in pig testes [31].

Another thermal stimulation-related cause of apoptosis during spermatogenesis is DNA damage [32]; heat affects the integrity of the sperm and leads to breaks in the DNA double strand. Heat stimulation altered chromosome structures and reduced chromatin material in rodents [33]. Furthermore, during meiosis, heat stress can also cause abnormal segregation of sex chromosomes and lead to the existence of unpaired Y chromosomes, which cause spermatocytes to undergo apoptosis [34]. DNA repair during spermatogenesis is essential for meiotic recombination and the repair of DNA damage in developing germ cells. Each testis has intact antioxidant-reducing capacity [35], involving a complex system with more than 130 genes related to gene protection. These gene proteins are involved in mismatch repair, nucleotide excision repair, basal excision repair, single-strand break repair, and double-strand break repair. Mismatch repair can fix small mismatches or loops. Nucleotide excision repair is primarily involved with reversing UV-induced and oxidative DNA damage. Basal excision repair mainly replaces abnormal (including oxidized) bases in DNA. However, many DNA repair genes, such as *Ogg1* (base excision repair), *Xpg* (nucleotide excision repair), *Rad51* and *Rad54* (double-strand break repair), were found to be down-regulated after heat treatment at 43°C [36]. In addition, there was reduced expression of polyADP ribose polymerase (PARP), which is involved in the detection of strand breaks and in signaling in base excision repair and nucleotide excision repair pathways [37]. A recent study found that deleted in azoospermia-like (DAZL) has a role in the fate of germ cells. The endogenous Dazl protein is essential in the formation of stress particles in sperm formation and sperm cell survival during local heat stress. The stress particles are cytoplasmic dense granules responding to multiple stress of environment, which contain polyadenylated mRNAs, 40S ribosomal subunits, translation initiation factors, and some RNA-binding proteins. The transient appearances of them can store, degrade, or reinitiate messenger ribonucleoprotein when heat stress occurs, and bring a fast recovery against the damage of heat.

It is widely believed that the production of reactive oxygen species (ROS) seems to be of utmost importance for apoptosis of germ cells and DNA damage [38]. ROS are molecules with at least one unpaired electron, making them highly unstable and extremely reactive for lipids, amino acids, and nucleic acids [39]. A 42°C heat treatment generated oxidative stress in the testisby the up-regulation of ROS and down-regulation of antioxidants [40]. In normal testes, ROS are maintained at an acceptable level due to the antioxidants, and when the balance between ROS and antioxidants is oxidative stress will ensue, then followed by apoptosis [41]). Heat stress induces up-regulation of heme oxygenase 1 (HMOX1) and antioxidant enzymes, namely glutathione peroxidase 1 (GPX1), glutathione S-transferase α and superoxide dismutase 1 (SOD1), indicating a robust oxidative stress response [42].

### High temperatures damage to BTB and Sertoli cells

Mature Sertoli cells are the main supporting cells of the spermatogenic epithelium and provide the necessary nutrients and support for developing spermatogenic cells. Sertoli cells are also an important component of the BTB [43,44]. They form the BTB by tightly connecting through basal ectoplasmic specialization [45]. The BTB is important for spermatogenesis and plays an important role in the physiology and pathology of testes [46]. An increase in testicular temperature can disrupt the function and morphology of the Sertoli cells [47], which in turn lead to the loss and infertility of the germ cells. Heat stress triggers reversible SC dedifferentiation and ultrastructural damage to the BTB due to changes in multiple BTB-associated proteins [48,49,50]. Thermal stimulation causes a decrease in the expression of two specific proteins in Sertoli cells, transferrin and Wilms’ Tumor 1 protein (a marker of Sertoli secretion and differentiation function), and loss of microfilament (f-actin) and microtubule network (α-tubulin) organization triggers a change in the cytoskeleton. After 48 h of short heat treatment (30 min) at 43°C, the mRNA and protein levels of the tight junction molecules occludin and ZO-1 of mice were significantly decreased, which caused loose tissue and high permeability in the BTB [51]. Metabolomic analysis revealed that nicotinic acid, carnitine, 2-hydroxypalmitic acid, adenosine monophosphate, niacinamide, creatine, and glutamine were affected by heat stress. Thermal stimulation also disrupted the mRNA levels of inflammatory cytokines IL-1α, IL-1β, and IL-6 and increased the expression levels of BTB factors, connexin 43, ZO-1, vimentin, claudin 1, and claudin 5 [52]. Androgen activities are mediated by the androgen receptor (AR), a member of the nuclear receptor superfamily. Upon androgen binding, AR translocates into the nucleus and interacts with various coregulators that function in a sequential and combinatorial manner to regulate the expression of AR-responsive genes [53]. But AR expression dramatically decreased after heat treatment, treatment of the monkey Sertoli cells with an AR antagonist, flutamide, could mimic the heat-induced changes in the expression of junction-associated molecules in Sertoli cells. Overexpression of AR in the Sertoli cells up-regulated the expression of BTB-associated protein. In summary, these results indicate that the decreased AR expression after heat treatment is involved in heat-induced cell junction disruption [50].

AMPK is a regulator of cellular energy metabolism and promotes the phosphorylation of downstream genes, regulating ATP biosynthesis and catabolism and inhibiting NADPH oxidase-derived ROS in mice to reduce lipopolysaccharide damage in the blood–brain barrier [54]. In the testes, the activation of AMPK consolidates tight junctions and promotes spermatogenesis [30,55]. In Sertoli cells, thermal stimulation inhibits the phosphorylation of AMPK, which down-regulates tight junction proteins, including claudin 11, and causes the excessive accumulation of ROS [56]. ROS regulate the cell cycle and the induction of apoptosis by the AMPK-signaling pathway and endoplasmic reticulum stress [57]. The continuous exposure of rat testicles to 43°C *in vitro* led to the rapid accumulation of neutral fat. Furthermore, the continuous thermal stimulation of Sertoli cell *in vitro* cultures increased the level of triacylglycerol and the accumulation of lipid droplets, and decreases mitochondrial lipid metabolism and fatty acid oxidation. These lipid changes indicate that high temperature affects the mitochondrial physiological state of Sertoli cells and causes ROS to induce depolarization of the membrane [58]. After thermal stimulation, Sertoli cell mitochondria degenerate, the smooth endoplasmic reticulum and intercellular space increase in size, lipid peroxidation increases. Above all, the protection of Sertoli cells toward spermatogenesis is incomplete [59].

Sertoli cells undergo changes under heat stress, but they have a certain resistance to thermal damage because changes in the BTB are reversible [60]. Studies have shown that musashi-1 (MSI-1) is essential in the construction of functional BTB structures and maintaining spermatogenesis, as MSI-1 knockdown *in vivo* disrupted BTB functional structure and spermatogenesis. When exposed to heat shock, the endogenous MSI-1 protein forms stress particles in Sertoli cells, which helps the cells resist environmental changes [61].

### Spermatogonial stem cells are resistant to hyperthermia

Although spermatogonial stem cells (SSCs) are affected by thermal damage, they can automatically recover, and heat stress rarely causes apoptosis of SSCs. Treatment at 43°C for 60 min did not lead to an increase in SSC apoptosis. Indeed, thermal damage has more of an effect on SSCs through non-apoptotic pathways. The study shows that heat treatment altered protein folding, protein localization, and self-renewal, and led to cell cycle arrest, but the expression of apoptosis-related genes did not change significantly [62]. However, there have been few studies on heat stress of SSCs, and it remains unknown whether there are other signaling pathways that confer heat stress resistance to SSCs.

### High temperatures affect hormone secretion by Leydig cells

Leydig cells are located in the Leydig parts of seminiferous tubules and are the main source of testosterone and other hormones [63]. The main function of Leydig cells is to secrete testosterone, which is regulated by the hypothalamus, pituitary, and itself, and ∼95% of testosterone in animals is secreted by Leydig cells [64]. Studies have reported that lipid deposition in mouse Leydig cells increased significantly after heat stress. Lipids are the raw material for the synthesis of androgens, and the observed lipid deposition suggests that testosterone synthesis was affected in the heat-shocked cells. Electron microscopy showed the nuclei of the Leydig cells were shrunken, the mitochondria and endoplasmic reticulum had expanded, the internal ridge structure had become unclear, the number of cells had decreased, and macrophages had infiltrated the surrounding area. Immunohistochemistry revealed that the number of Leydig cells with positive for testosterone were significantly reduced in scrotal hyperthermia groups than control group [65]. In addition, heat shock had significantly reduced the level of 3β-HSD expression, a key enzyme in the process of testicular synthesis and a functional marker of mature Leydig cells, and the concentrations of progesterone and testosterone. After the scrotum was shocked at 43°C for 15 min, there was no significant change in testosterone levels in the blood of the rats, while local testosterone levels in the testes decreased significantly on the second day after heat shock, and levels of blood follicle stimulating hormone (FSH) and luteinizing hormone (LH) appeared with significant change on the ninth day after heat shock. The change in testosterone levels indicates that heat shock damaged normal testosterone synthesis in rat Leydig cells but did not affect the circulating testosterone levels. In addition, heat stress affected individual hormone levels and reduced steroidogenic enzymes, Cyp11a1 and Hsd3b1, in Leydig cells. Expression of the activin gene was not affected at this time, but the concentration of the activin-binding protein follistatin increased more than two-fold. At 4–8 weeks post-treatment, the spermatids and early spermatids both recovered, but the elongating sperm cells were gradually lost, and most of the Sertoli cell genes had recovered; however, testicular activin A decreased and activin B increased. At week 8, serum inhibin was reduced, thus serum FSH increased. However, germ cell damage was not related to the obvious inflammatory response [66]. Heat stress can also induce endoplasmic reticulum stress and significantly affect the expression of steroidogenic enzymes and testosterone in Leydig cells [67]. Heat stress produce extracellular signal-regulated kinases 1/2 (ERK1/2) and JNK kinase and increases the protein and mRNA levels of MAP kinase phosphatase 1 (MKP-1). Phosphorylated MKP-1 induces ERK1/2 and JNK inactivation after heat stress. A study found that Leydig cells’ response to heat stress includes the activation of MAPKs related to cell survival (ERK1/2) and death (JNK) and the induction of an MAPK activity inhibitory loop [68].

## Methods of treating reproductive disorders caused by heat stress

Heat stress-induced testicular germ cell damage can be caused through oxidative stress, endoplasmic reticulum stress, changes to the body’s hormone expression, and disruption of the tight connections between cells. Therefore, different approaches may be needed to relieve reproductive damage caused by heat stress (Table 1).

**Table 1:** Reported strategies for treating testicular heat stress

Name of drug	Target genes	Type of cells	Function
Vitamin C [79]	HSP27 HSP70 HSP110 lipid peroxidation	Sertoli cells	Reducing oxidative stress and apoptosis, inducing HSP expression
Baicalin [88]	HSP70 caspase-3	Sertoli cells	Reducing apoptosis, increasing antioxidative enzyme activities
Ashitaba [54]	HSPa1a HSP40, HSPa11 HSPa2, GSS HO-1	Spermatid	Reducing oxidative stress, inducing HSP expression
Resveratrol [92,93]	IL-6 TNF-α	SSCs	Increasing testosterone secretion, reducing the inflammatory response, improving the antioxidant capacity
Korean RedGinseng [95]	GPx4 GSTm5 PRx4	Spermatid	Changes in the levels of antioxidant enzymes
Melatonin [40]	Jnk P38 MAPK HSP70	Sertoli cells	Alleviating autophagy, apoptosis, and oxidative stress in testis
None	clusterin mRNA	Spermatid Sertoli cells Leydig cells SSCs	Inducing HSP expression, reducing apoptosis
None	MK2	Spermatid	Inducing HSP27 and HSP70 expression

At present, there are many drugs that can alleviate reproductive damage caused by heat stress. The effects are differences in cell types and genes by different drugs. In general, they mainly use two cell signaling pathways: reducing the production of ROS and improving the resistance of cells to heat stress by regulating the expression of the HSP family.

### Mechanisms of self-repairing

#### ERK1/2

The ERK1/2 are evolutionarily conserved, ubiquitous serine-threonine kinases that regulate cellular signaling under both normal and pathological conditions by phosphorylating a variety of substrates. When heat stress starts, ERK1/2 induce different cascade reactions to regulate the level of cellular survival or apoptosis molecule, which protects part of male germ cells and somatic cells from being destroyed in testis. Study shows that 30-min heat stress can induce the enhance of pERK1/2 in immature boar Sertoli cells, which increase the level of HSP70 and then promote the production of lactate, the main substrate of ATP in developing germ cells, by accelerating glucose metabolism [69]. ERK can also make pachytene spermatocytes free from short-term heat stress-induced apoptosis by up-regulate metastasis-associated 1 against p53 [70].

#### Clusterin mRNA

Clusterin (CLU) is a stress-activated, ATP-independent molecular chaperone, normally secreted from cells. It plays important roles in protein homeostasis/proteostasis, inhibition of cell death pathways, and modulation of pro-survival signaling and transcriptional networks [71]. Clusterin can act directly or indirectly on apoptosis by regulating several intracellular pathways. These pathways include the oxidant and inflammatory program, BCL-2-associated X protein (BAX) pathway, BCL-2 antagonist of cell death (BAD) pathway, and MAPK pathway [72]. Following exposure to heat stress at 41°C for 12 h is significant increase in clusterin mRNA expression in rats’ Sertoli cells. However, after the treatment with siRNA targeting clusterin mRNA, the contains of clusterin mRNA lost 70% compared with controls at the end of above-mentioned stress, and the apoptotic index of Sertoli cells largely rises (the most prominent impact was observed within 24 h following heat treatment). These studies show that the cluster protein secreted by Sertoli cells can protect the testis from heat stress-induced damage [73].

#### Protein kinase MK2

It has been proved in murine GC-2spd cells that the protein kinase MK2 can activate the testis-enriched chaperone HSPA1L through site-specific phosphorylation of Ser^241^. The activated HSPA1L protects male germ cells from apoptosis due to heat stress; heat stress also activates the p38-MK2 stress signaling axis, which works together with HSPA1L to reduce the thermal stress-induced damage [74]. Interestingly, the first downstream gene of MK2 identified was another small heat shock protein (HSP), HSP27 [75], which forms multimers in the non-phosphorylated state but dissociates into monomers when phosphorylated by MK2 [76]. The monomeric HSP27 is involved in the polymerization of actin, causing cytoskeleton rearrangement, and promoting increased cell migration, which play an important role in the responses of cells to external stimuli [77].

### Additional medicine relieving heat stress

#### Vitamin C

Antioxidant compounds, such as vitamin C, can be used to relieve oxidative stress and mitigate injuries. Vitamin C is a very important water-soluble antioxidant because it can neutralize ROS in the water phase and act as an antioxidant before lipid peroxidation [78]. Studies have shown that mice Sertoli cells cultured *in vitro* can partly resist short-term heat stress via the prophylactic treatment of additional vitamin C. Doses of 20 μg/ml (*P*<0.01) and 50 μg/ml (*P*<0.01) significantly enhanced TM4 Sertoli cell viability under the heat stress treatment. Compared with controls, vitamin C pretreatment can reduce oxidative stress; induce the expression of HSP; prevent microtubule aggregation. Thereby the apoptosis of Sertoli cells caused by heat stress can be alleviated, and then the protection of BTB toward germ cells can be reconstructed. [79].

#### Melatonin

Melatonin (N-acetyl-5-methoxytryptamine) is a neuroendocrine hormone with strong antioxidant activities secreted by the pineal gland [80], and it is also synthesized in the testis [81]. Studies found that melatonin activates various antioxidant enzymes, scavenges free radicals [82], and protects the testes from inflammation [81]. Melatonin (20 mg/kg day) injection before hyperthermia in mouse has been reported to alleviate reproductive damage by inhibiting the apoptotic JNK and p38 MAPK signals to reduce apoptosis and oxidative stress. Post-treatment of melatonin suggests that the histological index of seminiferous epithelium, germ cells and testis in mouse have a clear recovery; and the tight junctions between Sertoli cells are also strengthened [40]. These results indicate melatonin can as a therapeutic drug for sub/infertility incurred by various testicular hyperthermia factors.

#### Betaine

Betaine (trimethylglycine, glycine betaine) is a zwitterionic quaternary ammonium antioxidant widely found in animals, plants, and microorganisms. Testes contain high levels of betaine and its transporter, members of the solute carrier six family. It has shown that betaine acts as a methyl donor to maintain male fertility, diminishes heat stress-induced damages of testicular germinal epithelium, and improves its regeneration by stimulating antioxidative defense of the testes. Metabolism of betaine leads to the production of S-adenosyl methionine (SAM), which is crucial for the creatine synthesis that is important for spermatozoon motility and function, as well as DNA, RNA, and histone methylation [83]. Study revealed that after 30 min of 42°C treatment, rats with betaine (250 mg/kg day) administration considerably up-regulates betaine-dependent metabolic pathways in the testes including creatinine biosynthesis, resulting in improvement of quantity and quality of epididymal spermatozoa as well as repair of germinal epithelium [84]. The study revealed the beneficial effect of betaine on improving epididymal spermatozoa in intact mice, as well as its potency to HS-induced complications of spermatogenesis.

#### Baicalin

Traditional herbs can also be used to mitigate the reproductive damage caused by heat stress. Many studies have confirmed that baicalin, a kind of flavonoid that is the main active ingredient in baicalensis, has many pharmacological activities [85]. Baicalin reduces cellular stress and apoptosis and increases BAX and BCL-2 expression by reducing HSP70 and caspase-3 [86,87]. Xanthamide, one of the functional components of baicalin, protects bovine Sertoli cells in *in vitro* cultures from acute heat stress damage by regulating the balance of TH1/TH2 cytokines Baicalin (50 mg/kg day) pretreatment effectively enhanced the activities of antioxidant enzymes and reduced the MDA content of mouse testes, which may partially explain the antioxidative effects of baicalin *in vivo*. In addition, baicalin pretreatment reduced the expression of P-JNK, FAS, FASL, caspase-9, caspase-3, and APAF-1; it has been deduced that baicalin inhibits the FAS/FASL apoptosis pathway in the testes of acute heat-stressed mice [88]. In summary, baicalin can alleviates these adverse effects of heat stress *in vivo*.

#### Angelica keiskei

The edible medicine Angelica keiskei (*Ashitaba keiskei*) (ashitaba), which originated in Japan, contains two active ingredients, xanthoangelol and 4-hydroxyderricin, which are the main polyphenol compounds of the plant which have anti-obesity, blood pressure lowering, and antidiabetic properties and other useful effects [89]. Ashitaba powder (AP; 57.5 mg/kg day) evidently prevented the decrease in HSPa11 and HSPa2 expression induced by short-term heat stress (15–20 min) in testicular cells of mice. The high expression of HSPa11 and HSPa2 in the testes is indispensable for fertility. AP may also reduce heat stress-induced generation of ROS by increasing glutathione synthase (GSS) and heme oxygenase-1 (HO-1) expression. The function of HSPs and antioxidant enzymes elevated by AP appeared to suppress the toxic effects of heat stress, including ROS generation [54]. Supplementation with AP can help prevent male infertility induced by heat stress.

#### Resveratrol

Resveratrol (3,4′,5-3,4′,5-trihydroxystilbene) is a natural non-flavonoid polyphenol compound mainly found in veratrum, polygonum cuspidatum, and grapes. Low concentrations of resveratrol can increase testicular sperm production in mice [90]. As a chaperone inducer, resveratrol activates heat-shock transcription factor 1 and accumulates Hsp70 protein at various mammalian, including human, peripheral cells. Resveratrol synergizes with mild to moderate heat shock and conferred cytoprotection against severe heat stress. Resveratrol activates heat-shock promoter-driven transcription, which decreases the temperature threshold of the heat-shock response, so the cells become more tolerant under more severe stresses [91]. Resveratrol (30 mg/kg day) was found to significantly improve the loss of SSCs and restore sperm production [92]. High-intensity exercise leads to a significant decrease in the amount of sperm and testosterone concentrations, leading to a reduction in spermatogenesis. Treatment with resveratrol (50 mg/kg day) was found alleviate the decline in spermatogenesis caused by high-intensity exercise [93]. In summary, Resveratrol might be an approach to promote SSC proliferation, cease SSCs loss iand improve the reproductive dysfunction of rats that was induced by heat stress.

#### Korean red ginseng

*Panax ginseng* Meyer, known as Korean red ginseng (KRG), is an important traditional herb used to boost the libido and improve male fertility [94]. A study has shown that rats fed with the extracts of KRG, ginsenoside Rg3, during the long-term heat stress were up-regulated the protein and mRNA levels of antioxidant enzymes closed to the normal in their testes, such as glutathione peroxidase 4, glutathione S-transferase μ 5 and peroxiredoxin 4. KRG (100 mg/kg day) counteracts the changes of these heat stress-induced antioxidase index in the testes, which improved the resistance of testis to the heat-induced oxidative stress, and enhances the testicular physiological function as well. Thus, the testis could provide a better environment condition to produce sperms [95]. KRG can be developed as an excellent therapeutic agent for hyperthermia-mediated male infertility.

## Discussion

Spermatogenesis is complex, continuous, and can be affected by various factors, such as disease, age, diet, and temperature. High temperatures can induce heat stress, which leads to a decline in sperm quality and fertility. Many factors can cause an increase in testicular temperatures, and these factors cannot be completely avoided; therefore, the ability of the testicles to regulate their temperature is crucial for healthy spermatogenesis. Heat stress occurs when the regulation capacity is exceeded and can cause germ cell damage and apoptosis, affecting male mammalian reproduction. However, mammals also use homeostasis mechanisms to resist heat stress; therefore, the molecular and genetic mechanisms of heat stress differ among different testicular germ cells. Heat stress mainly affects spermatocytes and spermatids and has the least effect on SSCs. When faced with high temperatures, spermatocytes and spermatids mainly undergo apoptosis, Leydig cells show changes in secretory capacity, and spermatogenic stem cells have trouble multiplying and cycling. Therefore, to relieve heat stress, different treatment methods are needed for the various cells in the testes.

Studies have shown that the reproductive damage caused by heat stress can be relieved by certain drugs and compounds, such as vitamin C, baicalin, ashitaba, resveratrol, melatonin, and others. These drugs target different cells and proteins in the testis, and most of them reduce cell damage by promoting HSP family expression and relieving oxidative stress. However, no drugs targeting MK2 or microRNA clusters have yet been reported, thus this area needs further study.

In general, using antioxidant drugs to treat heat stress is effective and feasible. Antioxidants act to inhibit the generation of oxidative stress, maintain cell homeostasis, and promote cellular resistance to the high-temperature environment. Regulating the expression of the HSP family or directly inhibiting the expression of apoptotic genes (caspase-3, BAX) can also alleviate heat stress to some extent, but this treatment method ignores the different effects of heat stress on the different cells of the testes. Significantly, high-temperature environments directly induce spermatocyte apoptosis but do not cause the apoptosis of SSCs or Leydig cells. Expression of the HSP family can increase the heat resistance of cells in the early stages of heat stress, but their continuous high expression can also have harmful effects. Therefore, future research should attempt to ascertain whether heat stress has similar effects on different testicular cells, such as oxidative stress, endoplasmic reticulum stress, and comprehensive mitochondrial dysfunction. If upstream factors of these biological response pathways can be inhibited, the generation of heat stress may be effectively treated and prevented. Understanding the causes and molecular mechanisms of testicular heat stress injury will help us to recognize and locate possible therapeutic targets for heat stress-induced reproductive damage in male animals.

## Abbreviations

AP: Ashitaba powder
APAF-1: apoptotic protease activating factor 1
AR: androgen receptor
BAX: BCL2-associated X
BCL-2: B-cell lymphoma-2
BTB: blood–testis barrier
DAZL: deleted in azoospermia-like
ERK1/2: extracellular signal-regulated kinases 1/2
FSH: follicle stimulating hormone
HSP: heat shock protein
HSPA1L: heatshock protein familyA member 1like
IL: interleukin
KRG: Korean red ginseng
MAPK: mitogen-activated protein kinase
MKP-1: MAP kinase phosphatase 1
MSI-1: musashi-1
ROS: reactive oxygen species
SC: spermatogonial cells
SSC: spermatogonial stem cell
ZO-1: zonula occludens-1
3β-HSD: 3β-hydroxysteroid dehydrogenase
